# Anthocyanin Biosynthesis Genes as Model Genes for Genome Editing in Plants

**DOI:** 10.3390/ijms22168752

**Published:** 2021-08-15

**Authors:** Emil Khusnutdinov, Anna Sukhareva, Maria Panfilova, Elena Mikhaylova

**Affiliations:** Institute of Biochemistry and Genetics, Ufa Federal Research Center RAS, Prospekt Oktyabrya 71, 450054 Ufa, Russia; emil.khusnutdinov.18@bk.ru (E.K.); ane4ka.suh@gmail.com (A.S.); masha.panfi@yandex.ru (M.P.)

**Keywords:** CRISPR, Cas9, Cas12, dCas, gRNA, PAP1, DFR, MYB, bHLH, WD40

## Abstract

CRISPR/Cas, one of the most rapidly developing technologies in the world, has been applied successfully in plant science. To test new nucleases, gRNA expression systems and other inventions in this field, several plant genes with visible phenotypic effects have been constantly used as targets. Anthocyanin pigmentation is one of the most easily identified traits, that does not require any additional treatment. It is also associated with stress resistance, therefore plants with edited anthocyanin genes might be of interest for agriculture. Phenotypic effect of CRISPR/Cas editing of *PAP1* and its homologs, *DFR*, *F3H* and *F3′H* genes have been confirmed in several distinct plant species. *DFR* appears to be a key structural gene of anthocyanin biosynthesis, controlled by various transcription factors. There are still many promising potential model genes that have not been edited yet. Some of them, such as *Delila, MYB60, HAT1, UGT79B2, UGT79B3* and *miR156*, have been shown to regulate drought tolerance in addition to anthocyanin biosynthesis. Genes, also involved in trichome development, such as *TTG1, GLABRA2, MYBL2* and *CPC*, can provide increased visibility. In this review successful events of CRISPR/Cas editing of anthocyanin genes are summarized, and new model genes are proposed. It can be useful for molecular biologists and genetic engineers, crop scientists, plant genetics and physiologists.

## 1. CRISPR/Cas Technology in Plant Science

The clustered regularly interspaced short palindromic repeats (CRISPR) system had been occasionally discovered in bacteria at the end of the XXth century, but only in the last 10 years was it applied for precise genome editing in mammalian cells and plants [1,2,3,4]. In 2020 this technique was recognized with the Nobel Prize in Chemistry.

Cas endonuclease and gRNA are essential parts of the system [5]. Cas9 nuclease is the most common in plant genome editing, however Cas12a (Cpf1), Cas12b (C2c1) and Cas12e (Cms1) show a lot of promise due to their ability to recognize T-rich PAMs (protospacer adjacent motif) and induce longer deletions. Unlike other nucleases, which were initially tested on animal cells, Cms1 was applied on plants first [6,7,8,9,10].

Catalytically dead Cas (dCas) is deprived of the ability to induce double strand breaks. Being fused to an effector, it can enhance or reduce gene expression, if targeted to a promoter region [11,12]. Nickases (nCas), which create single-strand breaks, are used for base-editing (creation of a precise single nucleotide polymorphism) [13] and prime editing (small programmed insertions and deletions) [14]. Longer insertions (knock-ins) can be made using the homology-dependent repair mechanism, which requires the presence of a donor DNA in the nucleus [15,16,17].

To create longer deletions for complete gene inactivation, multiple gRNAs can be used. Deletions predominantly occur between editing sites in a single gene [18]. Multiple gRNAs are also required for editing polyploid genomes, where several gene copies must be targeted simultaneously. Large expression cassettes with many gRNAs under the control of identical promoters were used in early approaches [19]. The invention of polycistronic gRNA expression cassettes allowed increasing the efficiency of multiplex gene editing in plants. In these systems multiple gRNAs, divided by either Csy4 sequence [20], tRNA [21] or ribozymes [9,22], are expressed by a single promoter. This technology is well developed and allows one to deliver up to 24 gRNAs [6].

Off-target mutations are an important problem in genome editing of mammals, because they pose a risk for an organism when they occur in a coding region. In plants off-target mutations are rare and can be segregated away by backcrossing. The majority of mutations happen during plant transformation and in vitro cultivation. Only one out of the twelve Cas9 sgRNAs resulted in off-target mutations, and no off-target mutations were detected after editing with Cas12a. More precise types of Cas, which require a longer PAM, or double nickases can further reduce the risk of off-target mutations. Conversely, this side effect can be used on purpose to target multiple gene copies containing SNPs [23,24].

The CRISPR market has been developing since 2016. The white button mushroom modified to resist browning was the first commercialized genome-edited organism [25]. Unlike traditional GM crops, predominantly herbicide and insect resistant, CRISPR plants are more environmentally friendly and sustainable [26]. For example, soybean oil with zero grams of trans fat was recently developed via CRISPR in the US [27]. Japanese scientists created a tomato rich in γ-aminobutyric acid (GABA) by deleting a C-terminal autoinhibitory domain of glutamate decarboxylase enzyme via CRISPR/Cas. GABA is believed to aid relaxation and help lower blood pressure [28]. The development of these new plant varieties was possible only because the nucleases and other important parts of genetic constructs were initially tested on model genes.

Nevertheless, CRISPR plants are still not wide distributed, which can be explained by public concerns about their safety. In the US plants that do not contain foreign DNA can be cultivated without passing through the regulatory process, but in Europe gene-edited crops are subject to the same regulations as transgenic plants. In most of the other countries their status is unclear [26].

Usually, gene editing requires a stable insertion of the T-DNA (including Cas, gRNAs and selection markers) in the plant genome [9,19,29]. As a result, edited plants are also considered genetically modified (GMOs) because they contain foreign DNA. The insertion can happen in a non-functional region, reducing the editing efficiency. If editing is successful, it can take years to get rid of the insertion.

“Clean” gene edited plants can be created using geminivirus-based vectors, first applied to perform a knock-in (insertion of a donor template) in plants [16,17,30,31,32,33]. Viruses such as Bean yellow dwarf virus (BeYDV) inside T-DNA initiate rolling circle replication of the CRISPR elements without integration into the genome [33]. To manage without integration of a foreign DNA, one should choose target genes that allow selection of editing events, for example, by affecting visible trait or increasing stress or herbicide resistance. Anthocyanin pigmentation is one of the most easily identified traits, that does not require any additional treatment. Many anthocyanin genes, such as *DFR*, *PAP1*, *ANT1*, *GLABRA2, TT8* are already used to test new CRISPR elements and genetic constructs. However, there are still many promising model genes that have not been used as CRISPR targets yet. In this review frequently used and potential targets for genome editing are summarized, and new model genes are proposed.

## 2. Anthocyanin Biosynthesis Pathway in Plants

**Figure 1:**
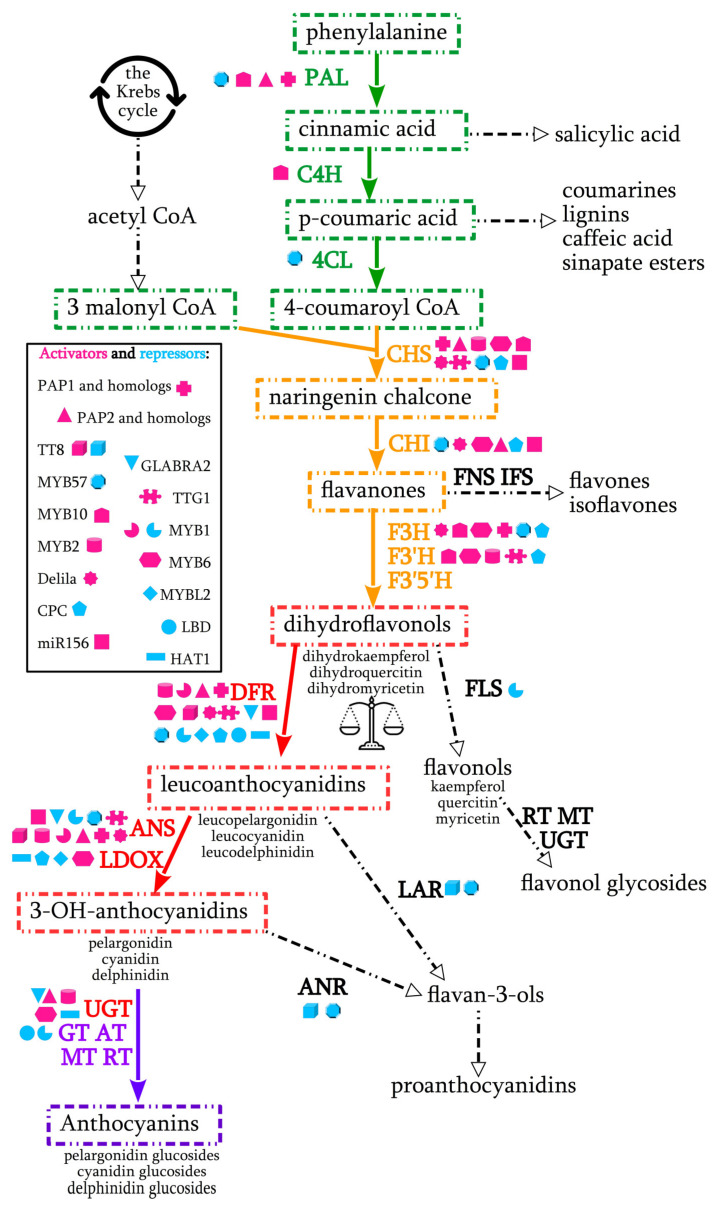
Scheme of anthocyanin pathway ant its transcriptional regulation. Early biosynthetic stage is highlighted in yellow, late biosynthetic stage is highlighted in red. Side branches are dashed. Enzyme names are abbreviated as follows: phenylalanine ammonia-lyase (PAL), cinnamate 4-hydroxylase (C4H), 4-coumarate:CoA ligase (4CL) chalcone synthase (CHS), chalcone isomerase (CHI), flavone synthase (FNS), isoflavone synthase (IFS), flavanone 3β-hydroxylase (F3H), flavonoid 3′-hydroxylase (F3′H), flavonoid 3′,5′-hydroxylase (F3′5′H), flavonol synthase (FLS), dihydroflavonol 4-reductase (DFR), leucoanthocyanidin reductase (LAR), anthocyanidin synthase (ANS), leucoanthocyanidin dioxygenase (LDOX), uridine diphosphate-dependent glucosyltransferase (UGT/UFGT), glucosyltransferase (GT), acyltransferase (AT), methyltransferase (MT) rhamnosyltransferase (RT) [38,39,40].

Anthocyanins are secondary metabolites, contributing to the red, purple and blue pigmentation in all tissues of higher plants. Anthocyanin biosynthesis pathway and all structural and regulatory genes involved in it are well studied in many plant species (Figure 1). Hyperaccumulation of anthocyanins is associated with greater resistance to herbivory [34,35], fungal deceases [36], bacterial infections [37], heavy metals [38] and other types of stress. Therefore, this trait is favorable not only because of its visibility, but also applicability in agriculture.

CHS, CHI, F3′H, FLS and F3H, involved at the early biosyntetic stage, lead to the production of flavonols and other flavonoid compounds. DFR, ANS and UGT are involved at the late stage [41,42]. CHS and DFR are the most specific for production of anthocyanins [43]. CHS initiates flavonoid biosynthesis. FLS, IFS and FNS lead to the accumulation of uncolored flavonoids (flavone and flavonol glycosides). Competition between FLS and DFR leads to either flavonol synthesis or anthocyanin accumulation [44]. Primary anthocyanidin aglycones (pelargonidin, cyanidin, delphinidin, peonidin, petunidin and malvidin) and genes responsible for their biosynthesis are conserved among plant species.

UGT, GT, RT, MT, and AT are responsible for glycosylation and acylation of anthocyanidin aglycones and production of various anthocyanins. There are over 500 unique anthocyanins and a great diversity of enzymes involved in anthocyanidin modifications [45]. For example, gentiodelphin [delphinidin 3-*O*-*β*-D-glucosyl-5-*O*-(6-*O*-caffeoyl-*β*-D-glucoside)-3′-*O*-(6-*O*-caffeoyl-*β*-D-glucoside)] is specific for genus *Gentiana* [46]. In gentiodelphin biosynthesis pathway 3-*O*-glucosylation is followed by the 5-*O*-glucosylation and 3′-*O*-glucosylation of delphinidin. 5-*O*-glycosyltransferase (*Gt*5*GT*), anthocyanin 3′-*O*-glycosyltransferase (*Gt3*′*GT*), and anthocyanin 5/3′-aromatic acyltransferase (*Gt*5*/3*′*AT*) contributed to violet, pink and mauve flower color shade in gentian [47]. Delphinidin 3-O-p-coumaroylrutinoside-5-O-malonylglucoside-3′5′-O-dihydroxycinnamoylglucoside (lobelinin), synthesized by UDP-rhamnose-dependent rhamnosyltransferase, provides the blue pigmentation in *Lobelia erinus* petals [48]. In general, the pathways for these late modifications are still relatively unexplored [49].

Transcription factors MYB, bHLH (MYC) and WD40 control the expression of late structural genes by binding to specific cis-acting elements in their promoter regions [41,50,51,52,53]. These three types of transcription factors form MBW complexes. For example, in *Arabidopsis thaliana*, complex of R2R3-MYB, bHLH, and WD40 proteins (TT2, TT8, and TTG1) activate the expression of proanthocyanidin genes [54,55]. MBW complex in petunia (*Petunia nyctaginiflora*) consists of AN2, AN1 and AN11 transcription factors [56].

Most of the anthocyanin biosynthesis repressors (MYB, LBD, HAT, NAC, etc.), also regulate other processes, such as trichome development, stomatal opening and fatty acid content.

Micro RNA miR828 triggers the cleavage of trans-acting small-interfering locus4 (TAS4) transcripts and negatively regulates anthocyanin biosynthesis [57,58,59].

In dicots and monocots anthocyanin biosynthesis is regulated differently, and there are also species-specific features [38]. For example, combination of MYB and bHLH transcription factors are required for anthocyanin biosynthesis regulation in Arabidopsis, but they can act alone in maize [60]. Moreover, each protein can be encoded by several genes, and genes can be represented by multiple copies. For example, in *A. thaliana* there are only 41 anthocyanin genes. However, 58 putative anthocyanin pathway genes are identified in *B. oleracea*, and 73 - in *B. rapa*, which experienced genome triplication. Among these genes, 67 are orthologs of 38 genes of *A. thaliana* [41,42].

Therefore, before editing an anthocyanin gene, it is important to study its role and copy number in the species of interest. Most of the predictions of anthocyanin gene functions are based on the expression profiling, however some genes were studied via generation of transgenic plants. The resulting data is important to choose proper targets for CRISPR/Cas. Several genes have already been used for genome editing multiple times, but the results were not always satisfying. Moreover, ectopic expression of the same gene in different varieties of the same species can result in accumulation of anthocyanins in various tissues and organs [61].

## 3. R2R3MYB Transcriptional Activators

R2R3MYB-domain subfamily of transcriptions factors is one of the largest in plants. R2R3MYB transcription factors include two imperfect repeats (R) domains. They usually bear an activator or repressor in the C-terminal end and regulate the development, defense response and production of secondary metabolites, including anthocyanins.

*Production of Anthocyanin Pigment 1* (*PAP1, MYB75*) and its homologs are the most frequently targeted MYB genes (Table 1). *PAP1* encodes R2R3 MYB transcription factor which predominates other MYBs in anthocyanin metabolism and is also involved in ROS scavenging. It is closely related to *AN2* gene of petunia, *MYB113-like* gene of carrot, paralog genes *ANT1*, *AN2-like* and *AN2* of tomato, *C1* and *p1* genes of maize and wheat, *IbMYB*1 of sweet potato, *LAP1* of *Medicago truncatula* [62,63]. Overexpression of *PAP1* gene results in purple coloration of a whole plant, determined by a single dominant allele. In *Arabidopsis PAP1* is induced by light, and upregulates the transcription of 38 anthocyanin genes, including *CHS, F3H, ANS* and *DFR* [40,43]. Upregulation of *PAL, CHS* and *DFR* by P1 transcription factor was reported in maize [64] (Figure 1).

Therefore, to increase anthocyanin pigmentation MYB transcriptional activators are to be overexpressed. There were several attempts to do it by targeting gene regulatory region upstream of the transcription start site, using Cas nuclease fused with transcriptional activators. Functional gRNAs for the promoter region of *PAP1* gene in *Arabidopsis* are already determined [86]. Despite a two- to seven-fold increase in *AtPAP1* mRNA content, changes in leaf color were not observed [29]. In another study, expression level of *AtPAP1* gene increased only two- to three-fold, but it resulted in the purple pigmentation of the leaves [12]. These differences may depend on cultivation conditions and the action of other transcription factors.

The insertion of constitutive CaMV 35S promoter before anthocyanin gene via CRISPR knock-in technique can also increase pigmentation. When BeYDV vector was used to deliver the donor template, gRNA and Cas9 cassette to target *SlANT1* gene of tomato via Agrobacterium-mediated transformation, dark purple plants were produced. All of them were heterozygous mutants. Homozygous mutants probably were not recovered because of the inhibitory effect of anthocyanins [16]. The experiment was successfully repeated using Cas12a nuclease and two guide RNAs [33]. 35S promoter-driven *An2* gene was used as a donor template to perform knock-in in tobacco, but this attempt was not successful [87].

CRISPR/Cas9-mediated knockout of *SlANT1* paralog, *SlAN2-like*, reduced anthocyanin content in tomato fruits. Out-of-frame mutants and mutant with one amino acid deletion were generated [65]. The CRISPR/Cas9 knockout of *SlAN2* in purple tomato cultivar ‘Indigo Rose’ resulted in a decreased anthocyanin content in vegetative tissues, however fruit color did not change [66]. The knockout of *DcMYB113-like* gene in purple cultivar of carrot using four gRNAs resulted in depigmentation. Heterozygous, biallelic, and chimeric mutants were produced [67]. Other varieties of carrot transformed with this gene, driven by the CaMV 35S promoter, demonstrated anthocyanin pigmentation of roots and petioles [88]. In transgenic wheat, overexpressing *ZmC1* gene of maize, pigmentation increased in the vegetative tissues such as coleoptiles, auricles, and stems [63].

*PAP1* is a promising target in many other plant species. This gene or its paralogs have been shown to increase anthocyanin accumulation in *Brassica oleracea* [89], rice [90], kiwifruit *Actinidia deliciosa* [91], wheat [92], *Freesia hybrida* [93], grape hyacinth (*Muscari armeniacum*) [94], *Lycium ruthenicum* and *L. barbarum* [95], and many others. It was also demonstrated that overexpression of *PAP1* gene promotes anthocyanin accumulation in hairy roots of different plant species [96].

*MYB90* (*PAP2*) belongs to the same subgroup with *PAP1* and probably originated from its tandem duplication. This gene also promotes anthocyanin biosynthesis in vegetative tissues by transcriptional up-regulation of the expression of structural genes [97]. However, it is rarely used as a target for CRISPR/Cas. *MYBA7* (*PAP2*) gene of grapevine (*Vitis vinifera*) was edited in order to combat grapevine diseases associated with anthocyanin accumulation. Mutants were predominantly bi-allelic, with 3 bp deletions or single bp insertions. Unfortunately, the authors did not evaluate changes in anthocyanin content or gene expression level [59].

When *MYB90/PAP2* gene of *A. thaliana* was introduced into tomato, anthocyanin content increased in all plant organs, but they were smaller in size and not fully purple [98]. Ectopic expression of *MYB90* gene from *Eutrema salsugineum* in tobacco and *A. thaliana* promoted anthocyanin accumulation in all organs, especially young leaves, and expression of *PAL*, *CHS*, *CHI*, *DFR*, *ANS* and *UFGT* genes [99].

Other MYB transctiptional activators were never used in CRISPR/Cas editing. The role and functions of some of them can be ambiguous in different plant species. For example, MYB1 (MYB114-like) transcription factor of onion (*Allium cepa*), radish (*Raphanus sativus*), mango (*Mangifera indica L.*) and apple (*Malus domestica*) induces anthocyanin production, but in strawberry (*Fragaria ananassa*) and lily it works as a repressor [100,101,102,103,104,105,106,107,108,109]. When *MYB1* gene was transiently repressed via RNA interference, anthocyanin pigmentation in onion decreased [100]. Ectopic expression of *MdMYB1* and *RsMYB1* in *Arabidopsis* and *MdMYB1* in cultured grape cells (*Vitis vinifera*) induced anthocyanin accumulation [103,110]. *RsMYB1* up-regulated six structural and two regulatory anthocyanin genes in *Arabidopsis*, including *TRANSPARENT TESTA8*, which encodes a bHLH transcription factor [91]. Co-expression of *RsTT8* and *RsMYB1*, as well as *MiMYB1* and *MibHLH2*, activated anthocyanin accumulation in tobacco leaves [105,110]. Expression of *RsMYB1a* in combination with *RsbHLH4* in the radish cotyledons and leaves also induced anthocyanin accumulation [104]. R2R3-MYB transcription factors PsMYB114L and PsMYB12L of *Paeonia suffruticosa* upregulated structural anthocyanin genes (*DFR* and *ANS*) and downregulated *FLS* gene, promoting the synthesis of anthocyanins instead of flavonols (Figure 1). Overexpression of these genes resulted in enhanced anthocyanin content in *Arabidopsis* leaves and apple calli [111]. *Myb1* gene in *Beta vulgaris* positively regulates the betalain pathway [112]. It was also discovered that genomic DNA of *RsMYB1a* gene is longer in red varieties of radish, therefore simple targeting of promoter region in green varieties may not be effective [65].

Therefore, *MYB1* gene cannot be used as a universal target for genome editing in many plant species. But it several species, such as onion and strawberry, where *MYB1* loss of function mutations have already been studied, this gene can be of some interest within the framework of CRISPR/Cas editing (Table 2).

*MYB2* gene also doesn’t act only as a transcriptional activator. In *Brassicaceae*, *Anthurium andraeanum*, *Dendrobium* hybrids orchid and purple celery (*Apium graveolens L.*) *MYB2* promotes anthocyanin accumulation, however in *Narcissus tazetta* and *Medicago truncatula* it acts as a repressor [118,119,120,121,141,142,143,144].

*Arabidopsis* expressing *MYB2* gene of *B. rapa*, *B. oleracea*, *A. graveolens* demonstrated increased anthocyanin pigmentation and up-regulation of early and late anthocyanin pathway genes, including *F3′H*, *DFR*, *UFGT*, *TT8*, *CHS*, *AN*S [118,119,120,121]. It has been suggested that purple pigmentation in *B. napus* may result from a single nucleotide and/or 2bp insertion in the promoter region of *BnaPAP2.A7*, an ortholog of *BoMYB2* of *B. oleracea* [122]. This knowledge can be used for creating precise mutations in *BnaPAP2.A7* and orthologous *MYB2* genes using prime editing.

Overexpression of *DcMYB6* gene of purple carrot (*Daucus carota*) in *Arabidopsis* enhanced anthocyanin accumulation in vegetative and reproductive tissues and upregulated structural genes *CHS, CHI, F3H, F3′H, DFR, LDOX* and *UGT78D2* [123]. Overexpression of *MYB6* of poplar (*Populus tomentosa*) increased accumulation of anthocyanins and proanthocyanidins but reduced secondary cell wall deposition [62]. Conversely, *MdMYB6* gene of apple inhibited anthocyanin biosynthesis in transgenic *Arabidopsis* [145].

Several other R2R3MYB transcription factors have been described as main activators of anthocyanin accumulation in certain organs of certain plant species. For example, in Asian pear (*Pyrus pyrifolia*), apricot (*Prunus armeniaca L.*) and apple (*Malus domestica*) *MYB10* gene is involved in the pigmentation of fruit skin [146]. Its ectopic expression in *Arabidopsis* resulted only in the pigmentation of immature seeds [114]. Conversely, in *Gerbera hybrida* ectopic expression of *MYB10* gene induced anthocyanin biosynthesis in undifferentiated callus, petioles, leaf veins and flower scapes. PAL, C4H, CHS, F3H and F3′H genes were also upregulated in transgenic plants [113]. In diploid woodland strawberry (*F. vesca*) and octoploid cultivated strawberry (*F.×ananassa*) *FaMYB10-2*, one of three *MYB10* homoeologs, was responsible for fruit color. CACTA-like transposon (*FaEnSpm-2*) insertion in the promoter of this gene was associated with enhanced expression and anthocyanin biosynthesis in strawberry fruits, however the presence of gypsy-transposon had the opposite effect [115]. A 8-bp insertion in the coding region of *FaMYB10-2* gene terminated protein synthesis and resulted in white fruit color [116]. An amino acid change caused by a single SNP in this gene resulted in the loss of fruit pigmentation [117]. Due to the small size of these mutations, *MYB10* is a suitable target for prime editing in strawberry (Table 2).

## 4. bHLH Transcriptional Activators

bHLH proteins can bind DNA either as a part of MBW complexes or individually. The N-terminal part of the bHLH usually interacts with MYB, and the C-terminal part interacts with WD40 [126].

*Transparent Testa8 (TT8)* acts as a positive regulator of anthocyanin biosynthesis, being sufficient for the expression of *DFR* and *ANS* genes [147]. TT8 is one of the key regulators of anthocyanin production in many plant species [42,60,148,149]. This gene also negatively regulates seed lipid accumulation through inhibiting the expression of *LEC1*, *LEC2*, and *FUS3* transcription factors and binding to the promoter region of genes involved in fatty acid biosynthesis. TT8 is the only bHLH transcription factor, involved in anthocyanin biosynthesis, ever subjected to CRISPR/Cas editing (Table 1). Unlike MYB transcription factors, it is represented in most of the species only by one copy, which makes it a noteworthy target. However, in a tetraploid *B. napus* there are two copies of *TT8* gene, located in A09 and C09 chromosomes. Yellow-seeded mutants with elevated seed oil and protein content and altered fatty acid composition were obtained by a knockout of both copies via CRISPR/Cas9. The mutation only in a single gene copy was not sufficient to recover yellow-seeded phenotype [68]. The mutation of two copies of *TT8* gene in tobacco (*NtAn1a* and *NtAn1b*) resulted not only in elevated protein and lipid content, but also in depigmentation of seeds and flowers. Expression level of *ANR* and *LAR* genes was decreased in these mutants [69].

It was demonstrated that natural mutations in *TT8* gene also result in yellow-seeded phenotype in *B. juncea* [150] and *B. rapa* [151]. *Arabidopsis TT8* mutant lacked red pigmentation in the leaves and seeds, which was restored by transformation with *RsTT8* gene of *R. sativus* [110].

In maize anthocyanin biosynthesis is controlled by ZmR transcription factor, belonging to the bHLH family. Transgenic wheat plants, overexpressing *ZmR* gene, accumulated anthocyanins in reproductive tissues, including seeds. Transgenic wheat plants with combined overexpression of *ZmC1* and *ZmR* genes accumulated the highest quantity of anthocyanins [63].

In transgenic tobacco bHLH transcription factor Delila of snapdragon (*Antirrhinum majus*) enhanced not only anthocyanin production in leaves and flowers, but also salt and drought tolerance (Table 2). Heterologous gene upregulated the expression of *CHS*, *CHI*, *F3H*, *DFR* and *ANS* genes in tobacco [124]. The simultaneous expression of AmDelila and R2R3 MYB transcription factor AmRosea1 activated the anthocyanin accumulation in the taproots [125]. The expression of the same two genes in *B. napus* promoted an increase in the anthocyanin content and the antioxidant activity in the transgenic plant leaves [126].

## 5. WD40 Transcriptional Activators

WD40 proteins upregulate anthocyanin and proanthocyanidin biosynthesis genes, but usually do not possess catalytic activity themselves. WD40 interact with bHLH transcription factors [152] or work in MBW complexes [153].

*Transparent Testa Glabra1* (*TTG1*, *An11* in petunia) is considered crucial for anthocyanin biosynthesis. In *A. thaliana* it is also involved in trichome and root hair development [154]. In loss of function *TTG1 Arabidopsis* mutants the seeds were depigmented, and trichomes were absent [155]. However, it has been shown that WD40 from *Medicago truncatula* does not affect trichome development. Loss of function mutants of *M. truncatula* did not accumulate mucilage. Synthesis of proanthocyanidins, flavonols, anthocyanins, and benzoic acid was reduced in their seeds [156]. In petunia, *An11* mutants demonstrated reduced pigmentation in the corolla not only due to lower anthocyanin content, but also modification of the vacuolar pH [56].

The knockout of *AtTTG1* via CRISPR/Cas9 genome editing resulted in pale seeds and in absence of trichomes in *Arabidopsis* leaves [70]. The *OsTTG1* CRISPR/Cas9 knockout mutant of rice demonstrated significantly decreased anthocyanin accumulation in various organs and reduction of trichomes in grains. *OsTTG1* protein could physically interact with *Kala4*, *OsC1*, *OsDFR* and *Rc* [71].

*AtTTG1* homolog *of Camelina sativa*, *CsWD40*, enchanced anhocyanin accumulation in green tea and tobacco and upregulated the expression of structural genes *CHS*, *F3′H*, *DFR* and *ANS*. The overexpression of this gene in *A. thaliana TTG1* loss of function mutant restored normal trichome and seed coat development. [154]. In apple TTG1 transcription factor interacted only with bHLH, but not MYB. It also didn’t bind to the promoter of *MdDFR* and *MdUFGT* genes. Ectopic expression of *MdTTG1* gene in *Arabidopsis* upregulated anthocyanin biosynthetic genes [152].

Therefore, the *TTG1* gene is a promising target in some, but not all plant species due to the visibility of associated traits: anthocyanin content and presence of trichomes (Table 1).

## 6. Transcriptional Repressors

However the knockout of a negative regulator of anthocyanin biosynthesis seems to be an easy way to increase pigmentation, transcriptional repressors are rarely used as targets in CRISPR/Cas approach.

Most of the transcriptional repressors of anthocyanin biosynthesis belong to MYB family [157]. Some have one R3 DNA binding domain, and other have two domains (R2R3). R2R3 transcription factors also possess EAR or TLLLFR repression motif at the C terminus, however R3 transcription factors have only DNA-binding domain, with the exception of MYBL2 [108].

Among repressors of anthocyanin biosynthesis, there are several proteins that belong to various families other than MYB. Their variable C-terminal region confers transcriptional control not only of structural anthocyanin genes, but also genes of transcription factors, involved in anthocyanin biosynthesis.

### 6.1. R2R3 MYB

R2R3 MYB transcription factors are described *A. thaliana* (AtMYB4, AtMYB60), *B. rapa* (BrMYB4), *Antirrhinum majus* (*AmMYB308*), petunia (*PhMYB27*), apple (MdMYB16 and MdMYB15L), banana (MaMYB4), grape (VvMYBC2-L1/3 and VvMYB4-like), strawberry (FaMYB1, FcMYB1), poplar (Ptr*MYB182* and Ptr*MYB57*), peach (PpMYB17-20), *Trifolium repens* (RED LEAF) and narcissus (NtMYB2) [97].

These transcription factors regulate the expression of different genes and can be divided in two groups: AtMYB4-like and FaMYB1-like repressors. AtMYB4-like type repressors act directly by binding to the MYB motifs in the promoters of structural genes. FaMYB1-like transcription factors act in MBW complexes, replacing positive MYB regulators. Difference in 12 residues of the DNA-binding domains between FaMYB1 and AtMYB4-like repressors may be responsible for the different types of interaction [103].

PtrMYB57 transcription factor of poplar forms MBW complexes with bHLH131 and PtrTTG1 and regulates the expression of *PAL4*, *4CL5*, *CHS*, *CHI*, *F3H*, *DFR1*, *ANS1*, *ANR1*, and *LAR1* structural genes. Among other repressors of anthocyanin biosynthesis, *MYB57* is the most noteworthy for being edited by CRISPR (Table 1). Loss of function mutants generated using CRISPR/Cas9 approach were characterized by a high anthocyanin and proanthocyanidin content. Overexpression of *PtrMYB57* gene reduced anthocyanin and proanthocyanidin content in transgenic poplar and suppressed structural genes [72]. Overexpression of the second repressor, *PtrMYB182*, in hairy roots and transgenic plants of poplar, gave the same result. *MYB182* inhibited transcriptional activation of anthocyanin biosynthesis pathway by a positive regulator, *MYB134*, and also downregulated shikimate pathway genes [158].

Other R2R3 MYB repressors have never been used as targets for CRISPR/Cas. Traditional approaches gave distinct results in different plant species. AtMYB4-like repressor of apple MdMYB16 inhibited the expression of *ANS* and *UFGT* genes directly binding to their promoter and reduced anthocyanin accumulation in apple calli [159]. Overexpression of its own *MaMYB4* gene in transgenic banana resulted in the significant decrease in anthocyanin content in leaves and pseudostems due to the inhibition of *CHS*, *ANS*, *DFR*, and *bHLH* expression [160]. However *AtMYB4* decreased the production of C4H, loss of function *Arabidopsis* mutants accumulated sinapate esters in their leaves instead of anthocyanins, and showed increased tolerance to UV-B irradiation [161].

*ANS, GT* and *DFR* genes were regulated by FaMYB1. This transcription factor suppressed accumulation of anthocyanins and flavonols in transgenic tobacco [103] and *Lotus corniculatus* [106]. Down-regulation of this gene in strawberry via RNA interference resulted in a significant increase in anthocyanin content [107]. RNAi-mediated silencing of *PhMYB27* gene in petunia increased anthocyanin content but shading of the plants prevented pigmentation. Transgenic petunia overexpressing this gene displayed reduced pigmentation in all tissues [162].

Overexpression of *AtMYB60* gene in purple lettuce resulted in depigmentation of the leaves [12]. However, the loss of function of this gene in *A. thaliana* and *V. vinifera* resulted in increased resistance to drought due to the reduction in stomatal openings without any phenotypic effect [163,164,165]. Therefore, among R2R3 transcriptional repressors, only downregulation of *PtrMYB57* and *FaMYB1* stably increased anthocyanin content (Table 1, Table 2).

Ability for transcriptional repression of anthocyanin biosynthesis can be achieved by a positive regulator due to the mutations. Two isoforms of a *B. napus BnaPAP2.A7* gene, lacking C terminal domain and a part of R3 repeat, lost the ability to interact with the bHLH protein and downregulated the expression of *PAL1*, *C4H*, *CHS*, *F3′H*, *MYB4*, *ANS* and *DFR* genes instead [122]. Overexpression of *MtMYB2*, which acts as a positive regulator in most of other species, reduced anthocyanin accumulation in hairy roots of *M. truncatula* and in *Arabidopsis* seeds. Anthocyanin accumulation increased in *M. truncatula MYB2* mutant [144]. *NtMYB2* repressed the transcription of structural anthocyanin genes, especially *UFGT*. Transient and ectopic expression of this gene in tobacco reduced the pigmentation [43].

### 6.2. R3 MYB

Among R3 MYB transcription factors, AtCPC and AtMYBL2 of *A. thaliana*, IlMYBL1 in *Iochroma*, PhMYBx of petunia, PtrRML1 of poplar, ROI1 of rose, GtMYB1R1 and GtMYB1R9 of *Gentiana triflora*, SlMYBATV and SlTRY of tomato [166] have been intensively studied. These transcription factors act as inhibitors of the MBW complexes [50,167].

MYBL2 is one of the most promising targets for CRISPR/Cas among them (Table 2). MYBL2-like repressors are closely related to R2R3-MYB and possess a part of an R2 domain and TLLLFR repressive domain. MYBL2 transcription factor binds to the bHLH protein GL3 and prevents the formation of MYB(PAP1/2)–GL3–TTG1 MBW complex. It also can bind to *BES1* and repress brassinosteroid controlled genes [130]. Purple varieties of *B. oleracea* lacked *BoMYBL2–1* coding sequences or had a substitution in the promoter region of this gene. It was demonstrated that these mutations alone were responsible for purple pigmentation [127]. Silencing of the *MYBL2* gene promoted anthocyanin accumulation of *A. thaliana* by increasing the expression of *DFR*, *LDOX*, *GL3*, *TT8*, and *PAP1* genes [51]. Two T-DNA insertion mutants of *Arabidopsis* demonstrated an increase in the anthocyanin content. Moreover, overexpression of *AtMYBL2* inhibited biosynthesis of proanthocyanidins [50] and prevented trichome initiation [128]. Expression of *IlMYBL1* gene from *I. loxense* in tobacco resulted in a loss of anthocyanin pigmentation [129].

*CPC* is also of interest as a target gene (Table 2), because it is not only involved in the repression of anthocyanin biosynthesis, but also acts as positive regulator of root hair formation and negative regulator of trichome formation [52,130,131]. It also interrupts the MBW activation complex by competing with the positive regulators PAP1 or PAP2. Overexpression of *CPC* gene in *A. thaliana* reduced anthocyanin accumulation and expression of DFR, LDOX, CHS, CHI, F3′H, and F3H genes. In loss of function mutants anthocyanin content increased in the presence of sucrose and under different stress conditions such as nitrogen depletion and continuous light [52,130]. Conversely, *ETC2*, *TRY*, and *CPC* triple CRISPR/Cas9 mutants had clustered leaf trichomes, while differences in pigmentation were not observed [168]. Therefore, special cultivation conditions may be required to achieve anthocyanin hyperaccumulation via editing of *CPC* gene.

### 6.3. Other Proteins

Unfortunately, regulation of anthocyanin biosynthesis by other proteins is poorly studied and confirmed only in a few plant species. However, most of the discussed genes are involved in stress response and may be of interest as targets to improve agricultural traits.

Lateral Organ Boundary Domain (LBD) transcription factors regulate plant development. Some of them negatively affect anthocyanin production. LBD37, LBD38, and LBD39 proteins repressed *PAP1*, *PAP2*, *TT8*, *MYB11*, *DFR*, *ANS*, *GT*, and *AT* genes and production of cyanidin in young leaves of *A. thaliana* (Figure 1). They are the best studied among non-MYB negative regulators of anthocyanin biosynthesis. Overexpression of these genes decreased anthocyanin production even under the action of stress (absence of N/NO_3_), while wild type plants accumulated anthocyanins. Loss of function of each gene resulted in an increased accumulation of anthocyanins without any stress treatment [53]. Overexpression of *MdLBD13* gene of apple in *A. thaliana* repressed anthocyanin accumulation and reduced nitrogen uptake [132]. In pear *Pyrus bretschneideri, PbrLBD20*, *PbrLBD35* and *PbrLBD53* genes were down-regulated in pigmented tissues, however *PbrLBD33* was up-regulated [169]. In *B. oleracea* upregulation of *LBD39* gene was associated with anthocyanin degradation, however *LBD39* and *LBD37* were down regulated in green tissues [133]. In purple cabbage the *LBD37* gene sequence contained 136 base pair insertion in the 2nd exon, resulting in alternative splicing and production of truncated proteins [136]. High expression level of *StLBD1-5* gene was suggested to decrease the accumulation of anthocyanins and drought resistance in potato [134]. Therefore, the functions of *LBD* transcription factors are conserved among plant species. Therefore, *LBD37*, *LBD38*, and *LBD39* can be recommended as universal targets for CRISPR/Cas editing (Table 2).

GLABRA2 is a member of the class IV homeodomain-leucine zipper (HD-ZIP) proteins. It has been successfully used as a model gene for CRISPR editing in *A. thaliana* [8,73]. It negatively affects the expression of *TT8*, *PAP1*, *PAP2*, *MYB113*, *MYB114*, *DFR*, *ANS*, and *UF3GT* genes (Figure 1). *GLABRA2* is antagonistic to R3 MYB genes, promotes trichome initiation and inhibits root hair formation. It also positively regulates mucilage biosynthesis in seeds and negatively affects seed oil content [74]. This gene was used as a model gene in development of germ-line-specific Cas9 system [73] and studies of CRISPR-Cas12a temperature sensitivity [8]. *GLABRA2* knockout mutants demonstrated trichome-less phenotype, however pigmentation remained unchanged. *GLABRA2* homolog in cotton, *GaHOX1* gene, also affected trichome development when overexpressed in *A. thaliana* [170]. However, in another study, anthocyanin accumulation was elevated in the loss-of-function mutant and reduced in the gain-of-function mutants [75]. Validation of these data require further studies of *GLABRA2*, especially in plant species other than *A. thaliana*.

HAT1 (HOMEOBOX *ARABIDOPSIS THALIANA*1) also belongs to HD-ZIP family and possess an N-terminal EAR motif. It participates in brassinosteroid signaling [171] and drought response [172]. Recently it has been demonstrated that this gene also negatively regulates anthocyanin biosynthesis. Loss-of-function mutants demonstrated enhanced drought tolerance and increased anthocyanin accumulation, whereas overexpression of *HAT1* repressed anthocyanin accumulation and decreased tolerance to drought stress. Transgenic *Arabidopsis* overexpressing *HAT1* gene accumulated five times less anthocyanins than control plants under intensive light and sucrose treatment due to the downregulation of *DFR*, *LDOX* and *UF3GT* genes (Figure 1). HAT1 interacted with MYB75, blocking the formation of MBW complex. Repression activity of HAT1 is supposed to be abolished through deletion or mutation of the EAR motif [140]. These data suggest that *HAT1* is a promising target for gene editing in *A. thaliana*. Unfortunately, homologs of this gene are not yet described in other plant species.

Members of NAC transcription factor family are involved in the response to abiotic stress. Overexpression of *NAC019* gene in *B. oleracea* reduced drought tolerance, repressed *PAL*, *C4H*, *CHS*, *F3H*, *ANS* and *UFGT* genes and decreased anthocyanin accumulation under drought conditions [173]. Under light stress, AtNAC078 of *A. thaliana* acted as a positive regulator of anthocyanin production, however AtNAC042 and AtNAC032 acted as negative regulators [174,175]. In apple *MdNAC52* gene promoted anthocyanin accumulation in apple calli by interacting with the promoters of *MdMYB9* and *MdMYB11* [176]. Transient expression of *LcNAC13* gene of litchi in tobacco leaves repressed anthocyanin genes *CHS*, *CHI*, *F3H*, *F3′H*, *DFR*, and *MYB1* by directly binding to their NAC*s* motifs and regulating their transcription [135]. NAC transcription factor JUNGBRUNNEN1 (JUB1) in *A. thaliana* downregulated the expression of *PAP1* and *PAP2*. Cyanidin derivative levels were decreased in transgenic 35S:JUB1 *A. thaliana* plants, while in loss of function mutants pigmentation did not change [177]. Overexpression of *AtJUB1* gene in tomato increased salinity tolerance, but changes in anthocyanin accumulation were not reported [178]. Therefore, NAC transcription factors act differently depending on the plant species and cannot be used as universal targets.

## 7. microRNAs

Micro RNAs are short non-coding small RNAs, which regulate gene expression via cleaving target mRNAs or preventing gene translation. In *A. thaliana* four miRNAs (miR156, miR165/166, miR828 and miR858) are involved in the regulation of anthocyanin biosynthesis.

Sequence of *miR828* is complementary to a region of *TAS4* and the MYB genes *MYB82* and *MYB113*. *TAS4* is cleaved by miR828 and produces small RNAs which are suggested to target either *MYB113*, *MYB75* and *MYB90* [58,179,180,181] or *PAP1*, *PAP2*, and *MYB113* [57,59]. As a result, miR828 reduces the expression of many structural genes in anthocyanin biosynthesis pathway in *Arabidopsis*, including *PAL*, *CHS*, *CHI*, *F3H*, *F3*′H, *DFR*, and *LDOX*. In transgenic *A. thaliana* overexpression of this miRNA reduced anthocyanin content [58].

miR828 is conserved in both dicot and monocot plants [58]. In apple peel *miR828* expression was reduced during rapid fruit coloration [182]. In lily, miR828 suppressed *MYB12* gene and produced bicolor patterns in lily flowers [183]. However, it is reported that in pigmented sectors of the tuber of purple potato *miR828* expression was higher than in unpigmented sectors [184]. In grape both miR828 and miR858 target anthocyanin repressor and promoter of flavonol biosynthesis *MYB114* [185].

Expression of *miR858* is usually associated with flavonol metabolism and susceptibility to cyst nematodes [186,187]. In *A. thaliana* miR858 downregulates MYB11, MYB12, and MYB111 transcription factors, which are considered responsible for the direct induction of early biosynthesis genes [41,188]. Overexpression of this *miRNA* in *Arabidopsis* significantly changed rosette size, flowering time, and metabolite content, however no difference in color was detected. When the effect of miR858 activity was decreased by artificial target mimic, the expression of *MYB* genes and the synthesis of flavonoids increased at the cost of lignin synthesis [79]. Another research showed that miR858a activated anthocyanin accumulation via inhibition of *MYBL2* [189]. In potato endogenous expression of *miR858* decreased flavonol content through repressing *MYB12* gene [186]. However, in kiwifruit overexpression of *miR858* inhibited anthocyanin biosynthesis [190]. Therefore, there are conflicting data on the role of *miR858* and *miR828* in plants.

MicroRNAs 165 and 166 differ by only one nucleotide. Overexpression of *miR165* in transgenic *Arabidopsis* disrupted the development of shoot apical meristems and promoted red pigmentation in the narrow cotyledons [191]. Structural anthocyanin genes were highly up-regulated in these transgenic plants. In radish *miR9748*, *miR870*, and *miR165a-3p* targeted the ETHYLENE INSENSITIVE 3 (EIN3) protein, which probably regulates the anthocyanin accumulation by mediating the sucrose signaling pathway [192]. In general, information on the role of *miR165/166* in anthocyanin biosynthesis is deficient. The importance of this micro RNAs for normal development of shoot apical meristems makes them inappropriate targets for the knockout.

MicroRNA miR156 targets *MYB11*, *MYB12*, and *MYB113* transcription factors and *Squamosa Promoter Binding Protein*-*Like* (*SPL*) genes in *A. thaliana* [193]. It was suggested that *SPL9* negatively regulates anthocyanin accumulation preventing the formation of MBW complexes. Increased expression of *miR156* in transgenic *Arabidopsis* promoted accumulation of anthocyanins, whereas reduction of *miR156* activity promoted synthesis of flavonols. However, the difference in pigmentation was hardly visible to the naked eye. When *miR156* of sweet potato was overexpressed in *A. thaliana*, anthocyanin content in plants visibly increased due to the upregulation of *CHS*, *CHI*, *DFR* and *ANS* [116,137]. Levels of anthocyanins, flavones, and flavonols were increased in transgenic poplar plants overexpressing *miR156*, however lignin content reduced [116]. Overexpression of a *miR156* gene of blueberry (*VcMIR156a*) in tomato enhanced anthocyanin biosynthesis and chlorophyll degradation in the stem [138]. In alfalfa (*Medicago sativa L.*) *miR156* positively regulated drought tolerance via interplay with *SPL13*, promoted anthocyanin accumulation and photosynthetic efficiency during drought stress [139].

Among all these micro RNAs, only miR156 stably acted as activator of anthocyanin biosynthesis in all studied plant species. Therefore, it can be used as a target for activation via knock-in or effector fused with Cas nuclease. Knockout of *miR156* can’t be recommended because resulting mutants can become more susceptible to drought.

## 8. Structural Genes of Anthocyanin Biosynthesis

Among structural genes, *DFR*, *ANS* and *UGT* are the most important because they are involved at the late stage of anthocyanin biosynthesis [41,42]. Dihydroflavonol reductase (DFR) catalyzes the stereospecific conversion of (2*R*,3*R*)-dihydroflavonols to (2*R*,3*S*,4*S*)-leucoanthocyanidins and competes with FLS for dihydroflavonol precursors. Competition between FLS and DFR results either in flavonol or anthocyanin biosynthesis. Anthocyanin production is usually activated by MBW complex and is targeted by the most of the described transcription factors [39,44]. *DFR* gene is one the most popular targets for CRISPR/Cas editing in different plant species.

The knockout of DFR-B locus in the Japanese morning glory (*Ipomoea nil*) using CRISPR/Cas9 resulted in anthocyanin-less white flowers [76]. In *DFR* knockout mutants of black rice anthocyanin content was lower than in control plants, and seeds were ocher instead of black. CRISPR/Cas9 genetic construct targeted bases 62–85 from ATG in the first exon [77]. dCas9:EDLL-S2:VPR activation of *DFR* gene in tomato and tobacco *N. benthamiana* increased gene expression level 400-10000 fold, however the phenotypic effect was not evaluated [79]. Deletion of a 1013 bp part of the *DFR* gene in tomato using CRISPR/Cas9 resulted in the reduction of anthocyanin pigmentation in regenerated plantlets. Knock in of the previously deleted *DFR* sequence restored anthocyanin pigmentation due to the recovery of a functional *DFR* gene [78]. Knockout of *DFR* gene was successfully accomplished in maize, but the phenotypic effects were not described [80].

Uridine diphosphate-dependent glucosyltransferases (UGTs) are involved not only in anthocyanin biosynthesis, but also in stress response. Overexpression of anthocyanin rhamnosyltransferases *UGT79B2* and *UGT79B3* in *Arabidopsis* increased anthocyanin accumulation and plant tolerance to cold, drought and salt stress. Loss of function mutants, generated by RNA interference and CRISPR-Cas9, were more susceptible to stress and had reduced anthocyanin content [81]. *UGT79B2* and *UGT79B3* can be considered as targets for CRISPR editing, however they are not yet described in many plant species.

Knockout of anthocyanin glycosyltransferase genes *Gt*5*GT* and *Gt3*′*GT* and acyltransferase gene *Gt*5*/3*′*AT* in blue-flowered gentian using CRISPR/Cas9 and two gRNAs resulted in large deletions and inversions between target sites. Loss of function of each gene resulted in a specific flower color shade due to predominance of different delphinidin derivatives [47]. However ornamental plants with different flower shades might be of commercial interest, radical change in color is required to use gene as a model for CRISPR.

Among early stage genes, *F3′*H and *F3H* were successfully edited via CRISPR/Cas with visible phenotypic effect. F3′H is necessary for formation of cyanidin type anthocyanins. Loss of function of this gene in *Arabidopsis* resulted in the production of kaempherol instead of quercetin [77]. The knockout of this gene in the red flowering poinsettia (*Euphorbia pulcherrima*) by CRISPR/Cas9 resulted in the change of bract color from red to yellow due to production of pelargonidin. Cyanidin content in the mutants decreased [82]. *F3*′H mutants of black rice with 11- and 22-base deletion in the coding region had the same depigmented ocher seed phenotype as *DFR* mutants. Anthocyanin content reduced from 41.9 to 2.5-4.0 mg/g of fresh weight [77].

The knockout of *F3H* gene of *Torenia fournieri* by CRISPR/Cas9 resulted in depigmentation of flowers [83]. Editing of this gene in carrot callus system produced white cells which could be visually distinguished from purple wild-type cells [84]. *F3H* CRISPR/Cas9 knockout mutants of the purple hypocotyl tomato resulted in depigmentation of hypocotyls [85].

Therefore, all described structural genes, except *GT*s and *AT*s, can be used as targets for stimulation of pigmentation. However, the preferred use of *DFR* gene as a target can be explained by small copy number. While some structural genes, such as *PAL*, have at least four copies even in *A. thaliana*, *DFR* and *F3’H* are usually represented by one copy. In tetraploid *B. napus* there are only two copies of each of these genes. *F3H* has three copies in *B. rapa* and four copies in *B. napus*. *CHS* gene is represented by one copy in *Arabidopsis*, parsley, and snapdragon, but in the majority of other species (petunia, ipomoea, legumes) chalcone synthase is encoded by a multigene family [194]. *DFR* gene is also a target of nearly all described transcription factors (Figure 1), which contributes to its predominant role in anthocyanin biosynthesis.

## 9. Conclusions

Anthocyanin genes are the most promising targets for validating efficiency of CRISPR/Cas vectors due to the visibility of the pigmentation to the naked eye. Visual assays can help to avoid the use of selective genes of antibiotic and herbicide resistance and reporter genes of fluorescent proteins and beta-glucuronidase.

Many of the described genes are involved not only in anthocyanin biosynthesis, but also in stress response. For example, *Delila*, *UGT* and *miR156* positively regulated drought tolerance. The knockout of *TT8* and *GLABRA2* genes increased seed oil content (Table 1). Therefore, produced plants can be of value to agriculture.

It is noteworthy that CRISPR/Cas editing of the same gene did not always give the same results [12,29]. It may happen due to the differences in genetic constructs, rtPCR primers and cultivation conditions. Illumination, sucrose and pH can significantly affect anthocyanin pigmentation [52,130]. Precise determination of the mutations and cultivation conditions, required for visual effect, can improve the evaluation of CRISPR/Cas editing efficiency. At the present time the phenotypic effect of successful editing often remains unstudied [59,79,80].

Among genes, already used in genome editing, *DFR*, *PAP1* and its homologs appear to be the most universal and conserved among plant species, and therefore suitable to be used as model genes (Table 1, Figure 1). They have been used as targets not only for the knockout, but also for activation and knock-in. Early biosynthetic genes *F3′H* and *F3H* are also noteworthy targets, however they have only been used for a knockout, resulted in reduction in anthocyanin pigmentation (Table 1). Phenotypic effect of CRISPR/Cas-mediated transcriptional activation of these genes have not been studied yet. DFR, F3′H and F3H are also noteworthy for having antagonist enzymes FNS, FLS and IFS, involved in the biosynthesis of other flavonoids (Figure 1). Change in the expression level of these genes can significantly affect the balance in the pathway and pigmentation of the plant tissues.

Genes of enzymes involved in the modification of anthocyanins such as *AT* and *GT* are incapable of changing pigmentation intensity and can only affect color shade due to the change in the proportion of multiple anthocyanins [47].

Function of positive anthocyanin biosynthesis regulators MYB2 and *miR156* have been verified in several plant species. Studies of the effect of both increased and decreased expression of these genes on anthocyanin accumulation gave promising results in all cases (Table 2). Therefore, MYB2 and *miR156* can be recommended to be used for genome editing in various plants.

Negative regulators of anthocyanin biosynthesis such as *MYBL2*, *CPC*, *LBD* and *HAT1* remain underestimated as CRISPR/Cas targets (Table 2). The knockout of anthocyanin biosynthesis repressor *MYB57* increased anthocyanin content in a single study on a single species [72], however the effect of the *GLABRA2* editing gave contradictory effect in *A. thaliana* (Table 1). Further studies on different plans species are required to adopt negative transcriptional regulators as model genes for CRISPR/Cas. Knockout remains the most accessible genome editing technology, however the possibilities to increase anthocyanin pigmentation in loss of function CRISPR/Cas mutants are largely unknown.

Cas9 is widely applied in plant genome editing, but temperature-tolerant and precise Cas12a and geminivirus-based vectors open new prospects in creation of transgene-free agricultural crops with valuable traits. Nevertheless, these approaches are not always successful and require further study. Verification of new CRISPR/Cas toolkits and genetic constructs on model genes, involved in anthocyanin biosynthesis, can speed up their practical application. Adoption of anthocyanin pigmentation as selective and reporter trait contribute to sustainability and environmental safety of genome editing.

## Figures and Tables

**Table 1:** CRISPR/Cas editing with phenotypic effects.

Gene	Organism	Function	Method of Genome Editing	Editing Events
*PAP1*	*A. thaliana*	MYB transcriptional activator of anthocyanin biosynthesis	activation	no changes in leaf color [29]; purple pigmentation of the leaves [12]
*ANT1*	*Solanum lycopersicum*	MYB transcriptional activator of anthocyanin biosynthesis	knock-in	dark purple plants [16,33]
*AN2*	*S. lycopersicum*	MYB transcriptional activator of anthocyanin biosynthesis	knockout	reduced anthocyanin content in tomato fruits [65]; decreased anthocyanin content in vegetative tissues [66]
*MYB113-like*	*Daucus carota*	MYB transcriptional activator of anthocyanin biosynthesis	knockout	depigmentation [67]
*MYBA7 (PAP2)*	*Vitis vinifera*	MYB transcriptional activator of anthocyanin biosynthesis	knockout	change in color were not evaluated [59]
*TT8*	*B. napus, N. tabacum*	bHLH transcriptional activatior of anthocyanin biosynthesis	knockout	depigmentation of seeds, elevated seed oil and protein content and altered fatty acid composition [68]; elevated protein and lipid content, depigmentation of seeds and flowers [69]
*TTG1*	*A. thaliana, Oryza sativa*	WD40 transcriptional activatior of anthocyanin biosynthesis	knockout	absence of trichomes and pale seeds [70], decreased anthocyanin accumulation in various organs, reduction of trichomes in grains [71]
*MYB57*	*P. nyctaginiflora*	MYB transcriptional repressor of anthocyanin biosynthesis	knockout	high anthocyanin and proanthocyanidin content [72]
*GLABRA2*	*A. thaliana*	homeodomain-leucine zipper repressor of anthocyanin biosynthesis	knockout	trichome-less phenotype [8,73]; anthocyanin accumulation and increased seed oil content in natural mutants [74,75]
*DFR*	*Ipomoea nil, O. sativa, S. lycopersicum, A. thaliana, N. benthamiana, Zea mays*	structural gene	knockout, activation, knock-in	reduced anthocaynin pigmentation [76,77,78]; knock-in of the deleted part of *DFR* gene restored gene function [78]; activation of DFR gene increased its expression level 400-10000 fold [79]; knockout was successful, but phenotypic effect was not evaluated [80]
*UGT79B2* and *UGT79B3*	*A. thaliana*	structural gene	knockout	mutants were more susceptible to stress and had reduced anthocyanin content [81]
5GT, 3′GT, 3′AT	*Gentiana triflora × Gentiana scabra*	structural gene	knockout	change in flower color shade due to predominance of different delphinidin derivatives [47]
*F3′H*	*Euphorbia pulcherrima, O. sativa*	structural gene	knockout	bract color changed from red to yellow [82], depigmented seeds and reduced anthocyanin content [77].
*F3H*	*Torenia fournieri, D. carota, S. lycopersicum*	structural gene	knockout	depigmentation of flowers [83], cells [84] and hypocotyls [85].

**Table 2:** Potential CRISPR/Cas targets with phenotypic effects.

Gene	Organism	Function	Method used to Verify Gene Function	Phenotypic Effect
*MYB10*	*A. thaliana, Gerbera hybrida, Fragaria vesca F.×ananassa*	MYB transcriptional activator of anthocyanin biosynthesis	generation of transgenic plants, SNP analysis	increased anthocyanin pigmentation in undifferentiated callus, petioles, leaf veins and flower scapes [113] and immature seeds [114]. Transposon insertions in the promoter region changed the fruit color [115]. An insertion in the coding region resulted in white fruit color [116]. An amino acid change caused the loss of fruit pigmentation [117].
*MYB1*	*Allium cepa*	MYB transcriptional activator of anthocyanin biosynthesis	RNA interference	anthocyanin pigmentation decreased [100].
*MYB1*	***F.×ananassa***	MYB transcriptional repressor of anthocyanin biosynthesis	RNA interference, generation of transgenic plants	down-regulation of this gene promoted an increase in anthocyanin content [107]; accumulation of anthocyanins and flavonols in transgenic tobacco was suppressed [103].
*MYB2*	*B. rapa, B. oleracea, B. napus A. graveolens*	MYB transcriptional activator of anthocyanin biosynthesis	generation of transgenic plants, sequencing	increased anthocyanin pigmentation [118,119,120,121]; mutation in promoter region of *BnaPAP2.A7* resulted in purple pigmentation in *B. napus* [122].
*MYB6*	*D. carota*	MYB transcriptional activator of anthocyanin biosynthesis	generation of transgenic plants	enhanced anthocyanin accumulation in vegetative and reproductive tissues [123].
*Delila*	*Antirrhinum majus*	bHLH transcriptional activatior of anthocyanin biosynthesis	generation of transgenic plants	enhanced anthocyanin production in leaves and flowers, salt and drought tolerance [124]; anthocyanin accumulation in the taproots [125] leaves [126].
*MYBL2*	*B. oleracea, A. thaliana, I. loxense*	MYB transcriptional repressor of anthocyanin biosynthesis	Sequence analysis of naturally purple plants, silencing, generation of transgenic plants	deletion of coding sequences or substitution in the promoter region were responsible for purple coloration [127]; loss of function promoted an increase in the anthocyanin content [50,51] and prevented trichome initiation [128]; expression of *IlMYBL1* gene in tobacco resulted in depigmentation [129].
*CPC*	*A. thaliana*	MYB transcriptional repressor of anthocyanin biosynthesis	generation of transgenic plants, loss of function mutants	positive regulator of root hair formation and negative regulator of trichome formation and anthocyanin accumulation [52,130,131].
*LBD37*, *LBD38*, and *LBD39*	*A. thaliana, B. oleracea, Malus domestica, Pyrus pyrifolia, S. tuberosum*	LATERAL ORGAN BOUNDARY DOMAIN repressors of anthocyanin biosynthesis	generation of transgenic plants, loss of function mutants, expression profiling	increased accumulation of anthocyanins in loss of function mutants [53,127]; repression of anthocyanin biosynthesis due to overexpression of LBD gene [132,133,134,135]; In purple cabbage pigmentation depended on an insertion in *LBD37* gene [136].
*miR156*	*A. thaliana, Ipomoea batatas, Populus alba ×P. tremula var. glandulosa, Vaccinium corymbosum, S. lycopersicum. Medicago sativa L.*	MicroRNA activator of anthocyanin biosynthesis	generation of transgenic plants	Increased expression of *miR156* promoted accumulation of anthocyanins [116,137,138,139]; reduction of *miR156* activity promoted synthesis of flavonols [137]; *miR156* positively regulated drought tolerance and photosynthetic efficiency [139].
*HAT1*	*A. thaliana*	homeodomain-leucine zipper repressor of anthocyanin biosynthesis	generation of transgenic plants	drought tolerance and anthocyanin accumulation increased in loss-of-function mutants and decreased in transgenic plants overexpressing *HAT1* gene [140]
